# Factors That Affect the Quality of Life of Mothers Caring for Children With Medical Needs at Home: Cross-Sectional Questionnaire Study

**DOI:** 10.2196/63946

**Published:** 2024-12-18

**Authors:** Kanako Nakamura, Yuko Hamada, Ayaka Fujita, Seiichi Morokuma

**Affiliations:** 1 Fukuoka Jo Gakuin Nursing University Koga City Japan; 2 Department of Nursing Faculty of Nursing Shimonoseki City University Shimonoseki Japan; 3 Cicely Saunders Institute of Palliative Care Policy & Rehabilitation, Florence Nightingale Faculty of Nursing, Midwifery & Palliative Care King's College London London United Kingdom; 4 Department of Health Sciences Graduate School of Medical Sciences Kyushu University Fukuoka City Japan

**Keywords:** home care, children with special health care needs, children with medical complexity, mother, quality of life, caregiver, questionnaire

## Abstract

**Background:**

The number of children requiring daily medical care is on the rise, with many being cared for at home. This situation places a significant burden on mothers, who often serve as the primary caregivers.

**Objective:**

This study aimed to clarify the factors that affect the quality of life of mothers with children who require home health care.

**Methods:**

A questionnaire study was conducted among mothers of children needing medical care at home, with 46 participants responding. The questionnaire included items regarding the child’s condition, the mother’s situation, and the World Health Organization Quality of Life-26scale.

**Results:**

Factors influencing the quality of life of mothers included whether the child attended daycare or school (β=.274; *P*=.04), the duration of home care (β=.305; *P*=.02), and the presence or absence of position changes (β=–.410; *P*=.003). The presence or absence of position changes had the most significant impact (adjusted *R*^2^=.327).

**Conclusions:**

The most significant factor affecting the quality of life of mothers of children requiring home medical care is the presence or absence of positional changes.

## Introduction

Children requiring medical care are those who, owing to medical advancements, continue to need medical care interventions such as suctioning secretions and tube feeding after long-term hospitalization in units like the neonatal intensive care unit. These children often rely on medical devices such as ventilators and gastrostomy tubes daily [1]. In the United States, children with special health care needs were defined in 1998 as those who have or are at increased risk for chronic physical, developmental, behavioral, or emotional conditions and require health and related services beyond what is generally required by children [2]. This definition includes children with congenital or acquired multisystem diseases, those with severe neurological conditions with significant functional impairments, and those who may require medical devices for daily life. These children are classified as having medical complexity and are medically fragile, with various intensive care needs that cannot be easily met by existing medical models [3].

Advancements in perinatal and neonatal medicine have increased the number of children whose lives are saved. In Japan, the number of children requiring medical care has doubled over the past decade, reaching 20,000 nationwide [1]. Many of these children, such as those using ventilators, are discharged directly from the neonatal intensive care unit to home [4].

The Ministry of Health, Labour and Welfare’s *Report on the Actual Conditions of Children Requiring Medical Care and Their Families* reveals that 94% of caregivers are mothers and 5.3% are fathers, indicating that mothers are primarily responsible for care. It also reports that numerous families are nuclear, suggesting that the caregiving burden is heavily concentrated on mothers [5]. These children often require not only life-sustaining medical procedures but also observation and care for symptoms unique to their condition to maintain stability, making it difficult for others to take over the mother’s role [6]. Consequently, mothers bear a significant burden and are often responsible for coordinating various social resources, including medical, welfare, educational, and public health services [6]. Despite insufficient support systems, families are primarily responsible for providing care. Matsui [5] found that the combined percentage of families who reported “agree” or “somewhat agree” to various lifestyle concerns was high: 71.1% for “chronic sleep deprivation,” 70.4% for “feeling strong anxiety about not knowing how long this will continue,” 69.7% for “unable to visit a medical institution when their own health deteriorates,” and 68% for “daily life is a continuous state of tension.” These high percentages indicate that their daily lives are continuously tense, sleep deprived, and anxious. This finding shows that mothers of children who require medical care experience significant caregiving and emotional burdens.

Furuya et al [7] have revealed that the quality of life (QOL) of parents of children with severe conditions tends to be generally low, influenced by the high caregiving burden at home. Senses Dinc et al [8] also reported that parents of children with Down syndrome have lower QOL than mothers of healthy children. They noted that the birth of a child with Down syndrome resulted in increased expenses and reduced participation in social and personal leisure activities, potentially affecting the mother’s QOL. In addition, factors that lower the subjective QOL of mothers of children with severe physical and intellectual disabilities at home include the child’s deteriorating health, decisions regarding treatment for secondary disabilities, the burden of dual caregiving for both parents and children, and anxiety over the mothers declining caregiving capacity. Conversely, factors that enhance subjective QOL include a sense of the child’s growth, social connections, fulfillment from reclaiming the mother’s social role, and family support during critical situations [9]. While there are scattered reports on the QOL of mothers of children with disabilities, few have specifically clarified the characteristics of the QOL of mothers of children requiring medical care. Comprehending these characteristics is crucial for providing support to improve their QOL. Therefore, this study had the following objective: to identify factors influencing the QOL of mothers of children requiring home medical care.

## Methods

### Study Participants

This study targeted mothers aged 20 years and older who had children (aged 3-12 years) requiring home medical care from late infancy to school age. To avoid imposing an excessive burden due to the home care situation and the child’s condition, mothers for whom the survey would be overly burdensome were excluded. In addition, mothers with multiple children requiring home medical care were excluded, as their sense of burden was expected to differ. Children who require medical care are often discharged from the hospital after about 6 months to a year, and in a survey of children who had been receiving home care for less than a year and a half, the majority of the children were aged between 1 and 3 years [10]. Based on this, we decided that the lower age limit of the children should be 3 years, as this is the age at which they would be familiar with childcare and home care. We also decided that the upper age limit of the children should be 12 years, as this is the age at which they would be in elementary school, based on their physical size and the school system.

### Recruitment

The study was conducted in Fukuoka Prefecture, Japan, a region with a population of 5 million, including an area with 1 million residents. The target population was children requiring medical care and their families who are supported by pediatric home care through home-visit nursing stations and clinics. The study content was elucidated to these facilities, which were then asked to select eligible mothers and distribute the questionnaires. The questionnaires were anonymous and self-administered, with respondents instructed to return them by mail. The survey period was from April to September 2016.

### Survey Items

This study was based on research into the factors that increase the burden of caring for a child [7], research into the factors that reduce QOL [8], and research into the factors that improve QOL [9]. The research framework was set up and the survey was conducted by focusing on the mother’s situation, including the sense of burden of child-rearing, and the child’s situation, including medical care, as factors related to the mother’s QOL.

The survey included the following: the child’s situation (age, disease, required medical care, daily life support, possession of a disability certificate, daycare or school attendance at nursery or regular school, and duration of home care) and the mother’s situation (age group, marital status, presence of cohabiting grandparents, presence of siblings of the child requiring medical care, occupation, household income, education level, and time spent caring for the child requiring medical care).

QOL was assessed using the Japanese version of the World Health Organization Quality of Life-26 (WHOQOL-26) [11], which has been verified for reliability and validity. The WHOQOL-26 defines QOL as “an individual’s perception of their position in life in the context of the culture and value systems in which they live and in relation to their goals, expectations, standards, and concerns.” While there are various scales with high reliability and validity for measuring QOL, this study adopted the concept of QOL, defined by the World Health Organization (WHO) as “a state of complete physical, mental, and social well-being and not merely the absence of disease or infirmity.” Therefore, the WHOQOL-26 was used, aligning with this interpretation. The WHOQOL-26 consists of 26 items, including 24 items across 4 domains (physical, psychological, social relationships, and environment) and 2 items that ask about the overall QOL. It seeks subjective judgments from the respondents. The physical domain includes activities of daily living, dependence on medication and medical aids, energy and fatigue, mobility, pain and discomfort, sleep and rest, as well as work capacity. The psychological domain includes body image, negative feelings, positive feelings, self-esteem, spirituality, religion, personal beliefs, thinking, learning, memory, and concentration. Social relationships include personal relationships, social support, and sexual activity. The environmental domain encompasses financial resources, freedom, physical safety and security, accessibility, quality of health and social care, home environment, opportunities to acquire new information and skills, participation in and opportunities for recreation and leisure activities, living environment, as well as means of transport. The respondents were asked to reflect on their circumstances over the past 2 weeks. Responses are given on a 5-point Likert scale ranging from 1 (not at all) to 5 (very much). Higher scores indicated a higher QOL. Cronbach α for the WHOQOL-26 ranged from 0.66 to 0.84.

### Statistical Analysis

Participant and child attributes, children’s medical care, and WHOQOL-26 scale scores were analyzed using the Mann-Whitney *U* test. The correlation between the caregiving burden scale and WHOQOL-26 was analyzed using Spearman rank correlation analysis. A multiple regression analysis was conducted for items exhibiting significant differences. The multiple regression analysis confirmed no multicollinearity between the independent variables, with no variables having a variance inflation factor exceeding 5. The statistical software used for these analyses was JMP Pro 15 (JMP Statistical Discovery LLC).

### Ethical Considerations

This study was conducted with the approval of the Clinical Research Ethics Review Committee of the Regional Department of Medical Care, to which the author was affiliated (2019-191). Participation in the questionnaire survey was voluntary and anonymous, and consent was obtained through the completion and return of the questionnaire.

## Results

### Participant Attributes

Table 1 shows the characteristics of the participants. Questionnaires were distributed to mothers providing home care to children requiring medical care. Responses from those not providing medical care (including bedridden care) or from grandparents were excluded, resulting in 46 mothers being included in the analysis. The average age of the 46 children was 5.8 (SD 2.3) years. Children’s conditions varied widely, with epilepsy (n=19, 41%) and cerebral palsy (n=16, 35%) being the most common. In addition, 23 (50%) children had two or more conditions, indicating that numerous children had multiple medical issues. Regarding disability certificates, 45 (98%) children had an identification booklet for individuals with physical disabilities, which was issued when a person was judged to have a certain level of physical disability and enabled access to welfare services, such as medical expense subsidies, tax benefits, and discounts on public transportation. In addition, 34 (74%) children had an identification booklet for individuals with intellectual disabilities, which enables access to similar welfare services as the identification booklet for individuals with physical disabilities. Numerous children have obtained both certificates.

**Table 1:** Participant attributes (N=46).

Participant attributes		Value, n (%)
**Mother’s age group (years)**		
	20s	5 (11)
	30s	23 (50)
	40s	18 (39)
**Marital status**		
	Married	42 (91)
	Other	4 (9)
**Siblings**		
	Yes	37 (80)
	No	9 (20)
**Cohabiting grandparents**		
	Yes	9 (20)
	No	37 (80)
**Occupational status**		
	Yes	11 (24)
	No	35 (76)
**Household income (JP ¥10,000^a^)**		
	≤400	15 (33)
	400 or more	29 (63)
	Missing data	2 (4)
**Child’s age (years)**		
	3-5	22 (48)
	6-9	23 (50)
	Missing data	1 (2)
**Daycare or school attendance**		
	Yes	35 (76)
	No	10 (22)
	Missing data	1 (2)
**Home medical care period (years)**		
	<5	20 (43)
	5 or more	26 (57)
	Missing data	2 (4)
**Hospitalization**		
	Yes	19 (41)
	No	27 (59)

^a^US $1=JP ¥105 on average from April to September 2016.

### Characteristics of QOL

#### Attributes of Mothers and Children and Their QOL

Table 2 shows the attributes of research participants and children, and WHOQOL-26 scores. Analysis of the attributes of mothers and children and their QOL revealed significant disparities in QOL scores based on certain attributes. Significant disparities were observed between marital status and social relationships QOL (*P*=.03); between children’s age and physical QOL (*P*=.007); between daycare or school attendance and WHOQOL-26 score (*P*=.03) and environmental QOL (*P*=.04); between duration of home care and WHOQOL-26 score (*P*=.005), physical QOL (*P*=.007), and environmental QOL (*P*=.007); and between psychomotor developmental delay and physical QOL (*P*=.04). The WHOQOL-26 scores were significantly higher for the group with daycare or school attendance (*P*=.03) and for those with a home care duration of less than 5 years (*P*=.01). Physical QOL was significantly higher in children aged 3-5 years (*P*=.007), those with a home care duration of less than 5 years (*P*=.007), and those with psychomotor developmental delay (*P*=.04). Environmental QOL was significantly higher in the group with daycare or school attendance (*P*=.04) and in those with a home care duration of less than 5 years (*P*=.007).

**Table 2:** Attributes of research participants or children and WHOQOL-26^a^ scores (Kruskal-Wallis test for mother’s age group and Mann-Whitney *U* test for others).

Attributes			Participants (N=46), n (%)		WHO QOL-26 score	*P* value			Physical QOL^b^			*P* value			Psychological QOL			*P* value			Environmental QOL		*P* value			Social relationship QOL			*P* value			Overall QOL (2 items)			*P* value	
**Mother’s age group (years)**							.50						.21						.77					.47						.70						.54
	20s	5 (11)		3.31				3.51						3.20						3.25					3.27						3.20					
	30s	23 (50)		3.15				3.20						3.27						2.96					3.49						2.80					
	40s	18 (39)		3.03				2.99						3.12						2.92					3.46						2.75					
**Marital status**							.89						.61						.54					.49						.03						.80
	Married	42 (91)		3.12				3.14						3.22						2.96					3.50						2.82					
	Other	4 (9)		3.11				3.29						3.04						3.09					3.00						2.88					
**Siblings**							.87						.90						.38					.11						>.99						.91
	Yes	37 (80)		3.10				3.13						3.24						2.91					3.45						2.82					
	No	9 (20)		3.20				3.23						3.08						3.25					3.48						2.83					
**Cohabiting grandparents**							.39						.75						.42					.45						.04						.53
	Yes	9 (20)		2.99				3.10						3.06						2.82					3.22						2.72					
	No	37 (80)		3.15				3.16						3.24						3.01					3.51						2.85					
**Occupation**							.28						.69						.40					.18						.83						.55
	Yes	11 (24)		3.28				3.29						3.33						3.15					3.45						2.95					
	No	35 (76)		3.07				3.11						3.16						2.92					3.46						2.79					
**Household income (JP ¥10,000^c^)**							.71						.09						.42					.60						.27						.22
	≤400	15 (34)		3.15				3.32						3.29						2.87					3.33						3.00					
	400 or more	29 (66)		3.07				3.03						3.12						2.98					3.51						2.69					
**Child’s age (years)**							.11						.007						.54					.31						.31						.22
	3-5	22 (49)		3.23				3.36						3.29						3.04					3.53						2.93					
	6-9	23 (51)		2.98				2.91						3.10						2.88					3.39						2.70					
**Daycare or school attendance**							.03						.22						.25					.04						.14						.54
	Yes	35 (76)		3.17				3.18						3.25						3.06					3.50						2.86					
	No	10 (22)		2.87				2.97						3.00						2.58					3.30						2.65					
**Home medical care period (years)**							.005						.007						.07					.007						.35						.10
	<5	20 (43)		3.32				3.43						3.40						3.23					3.54						2.97					
	5 or more	26 (57)		2.97				2.93						3.50						2.79					3.39						2.60					
**Hospitalization**							.14						.10						.15					.68						.13						.30
	Yes	19 (41)		3.24				3.02						3.09						2.95					3.36						2.72					
	No	27 (59)		3.03				3.33						3.36						3.02					3.60						2.97					
**Psychomotor developmental delay**							.06						.04						.15					.051						.41						.55
	Yes	10 (22)		3.37				3.49						3.45						3.27					3.50						2.90					
	No	36 (78)		3.05				3.06						3.14						2.89					3.44						2.81					
**Multiple diseases**							.40						.89						.59					.46						.79						.27
	Yes	21 (46)		3.16				3.17						3.23						3.03					3.48						2.98					
	No	25 (54)		3.09				3.13						3.19						2.93					3.44						2.70					

^a^WHOQOL-26: World Health Organization Quality of Life-26.

^b^QOL: quality of life.

^c^US $1=JP ¥105 on average from April to September 2016.

#### Medical Care for Child and Mother’s QOL

Children’s medical care and mothers’ QOL are represented in Table 3. The Mann-Whitney *U* test was conducted to examine the relationship between the medical care situation of the children and the QOL of the mothers. Significant disparities were observed in the WHOQOL-26 scores, with the group not requiring position changes scoring higher than the group that did (*P*=.003). Among the various aspects of medical care, the presence or absence of positional changes exhibited the most significant disparities in QOL (from *P*=.02 to *P*=.003).

**Table 3:** Medical care for child and mother’s QOL^a^ (Mann-Whitney *U* test).

Medical care		Participants (N=46), n (%)	WHOQOL-26^b^	*P* value		Physical QOL		*P* value		Psychological QOL		*P* value	Environmental QOL		*P* value		Social relationship QOL		*P* value	Overall QOL (2 items)		*P* value	
**Ventilator use**					.80				.31			.39				.87			.47				.41
	Yes	8 (17)	3.14			3.00				3.36			2.96				3.58			3.06			
	No	36 (78)	3.11			3.16				3.18			2.99				3.42			2.76			
**Tracheotomy site care**					.87				.60			.28				.82			.09				.94
	Yes	14 (30)	3.17			3.07				3.37			3.02				3.64			2.82			
	No	30 (65)	3.09			3.16				3.14			2.97				3.36			2.82			
**Oxygen inhalation**					.28				.21			.28				.35			.86				.16
	Yes	15 (33)	2.99			2.95				3.05			2.91				3.47			2.60			
	No	30 (65)	3.18			3.23				3.28			3.02				3.44			2.93			
**Inhalation**					.08				.002			.22				.36			.67				.06
	Yes	31 (67)	3.03			2.95				3.14			2.93				3.48			2.68			
	No	13 (28)	3.32			3.55				3.39			3.11				3.36			3.15			
**Aspiration**					.58				.22			.45				.67			.57				.51
	Yes	31 (67)	3.08			3.08				3.15			2.96				3.48			2.79			
	No	14 (30)	3.20			3.28				3.30			3.05				3.38			2.89			
**Position change**					.003				.003			.03				.02			.07				.19
	Yes	23 (50)	2.92			2.88				3.02			2.81				3.30			2.65			
	No	21 (46)	3.34			3.46				3.47			3.16				3.62			2.98			
**Care for gastrostomy or enterostomy**					.81				.12			.77				.89			.42				.97
	Yes	14 (30)	3.09			2.94				3.25			3.02				3.55			2.79			
	No	28 (61)	3.14			3.24				3.22			2.98				3.39			2.80			
**Feeding tube**					.08				.004			.40				.24			.71				.78
	Yes	29 (63)	3.02			2.94				3.14			2.92				3.43			2.79			
	No	14 (30)	3.31			3.53				3.34			3.14				3.48			2.80			

^a^QOL: quality of life.

^b^WHOQOL-26: World Health Organization Quality of Life-26.

#### Factors that Affect WHOQOL-26 scores

Table 4 shows the predictors of QOL in mothers of children with medical care.

**Table 4:** Predictors of QOL^a^ in mothers of children with medical care^b^.

Variable	β	*t*	*P* value
Presence or absence of daycare or school attendance	.274	2.13	.04
Home medical care period (2 groups)	.305	2.33	.02
Presence or absence of position change	–.410	–3.16	.003

^a^QOL: quality of life.

^b^*F*=7.79 (*P*<.001); adjusted *R*^2^=.327.

The significant factors influencing the WHOQOL-26 scores were examined based on the scores, attributes of the mothers and children, and the presence or absence of medical care, considering previous research findings. Three independent variables—daycare or school attendance, 2 groups of home care periods, and the presence or absence of position changes—were entered into the multiple regression analysis, with the WHOQOL-26 scores as the dependent variable. The variance inflation factor values of the independent variables were all below 5, indicating no multicollinearity. The presence or absence of daycare or school attendance (*P*=.04), home care periods (*P*=.02), and the presence or absence of position changes (*P*=.003) all had a significant impact on WHOQOL-26 scores. Position changes, home care periods, and the presence or absence of daycare or school attendance influenced the WHOQOL-26 scores, with position changes having the greatest impact.

## Discussion

### Principal Findings

In this study, we used the WHOQOL-26 scale to examine the QOL of mothers caring for children who require medical care at home. Standard data for the WHOQOL-26 scale were obtained from random population samples [11]. Compared with these standard data, the WHOQOL-26 scores of mothers caring for children requiring medical care at home were lower than those of the general population.

### Factors Related to the QOL of Mothers of Children Requiring Medical Care at Home

Multiple regression analysis identified factors influencing the WHOQOL-26 scores, including the presence or absence of daycare or school attendance, duration of home care, and presence or absence of position changes. The following section examines these factors.

### Daycare or School Attendance

The WHOQOL-26 scores were significantly higher in the group with daycare or school attendance. Daycare or school attendance provides not only connections between the family and health care providers but also social connections with families of similar children and educators. These factors are believed to contribute to improvements in maternal QOL.

Matsuzawa et al [12] cited a “Lack of opportunities to seek advice or obtain information” as one of the challenges experienced by parents of children requiring medical care. They reported limited interactions with other family members and insufficient opportunities for consultation with their children. Daycare or school attendance contributes to obtaining opportunities for advice and information, as well as alleviating challenges through interactions with other families. It is, therefore, considered a significant contributing factor to enhancing maternal QOL.

Furthermore, through daycare or school attendance, children can receive appropriate therapy and education to promote their growth and development. In addition, there have been reports [13] of children wishing to experience as many external activities as possible while staying healthy, indicating the importance of parents in promoting their children’s growth and development through experiences outside the home. Daycare or school attendance encourages children’s growth and development, resulting in mothers feeling satisfied with their children’s growth and development, thus enhancing their parenting satisfaction. Furthermore, through daycare or school attendance, parents may receive advice from experts on signs of growth that are not easily noticed under normal circumstances, leading to joy for parents and contributing to the enhancement of maternal QOL.

### Duration of Home Care

During home care, the WHOQOL-26 scores were significantly higher in the group with less than 5 years of care. Parents of children requiring medical care face additional burdens beyond typical childcare, including the technical aspects of medical care and various challenges, leading to a more demanding situation. Nygård and Clancy [14] reported, "When the burden of care becomes overwhelming, parents may lose motivation to continue caregiving, potentially leading to a decline in their caregiving abilities. This may affect the health of parents, family functioning, and the potential health status of children with illnesses." As reported, this suggests that longer care periods may be associated with various impacts and a potential decrease in WHOQOL-26 scores in the group with care periods of five years or more. This finding is consistent with the results of this study. Moyes et al [15] reported that the need for support among parents of children requiring complex medical care changes over time.　As the duration of a child's home care increases, it is not that mothers' needs for support disappear, but rather that their needs change over time and they continue to seek support. In this study as well, it is considered that insufficient support tailored to the needs of mothers with longer home care periods may have contributed to the decline in WHOQOL-26 scores.

### Position Changes

In the context of caring for children examined in this study, a significant association was found between the necessity of positional changes and the QOL of mothers. This association was substantial and treated with the same importance as other medical care items in the study.

Matsui and Takada [16] reported that managing artificial ventilation, aspiration, and injections for children with severe disability during the night increased mothers’ caregiving burden. However, they did not find significant disparities in assistance with excretion or position changes, suggesting that these tasks were perceived as extensions of child-rearing. In this study, position changes exhibited a significant association with mothers’ QOL, likely because, unlike typical child-rearing tasks that diminish as children grow, the need for positional care persists. In addition, as children’s bodies change, meticulous care for contractures, fractures, and pressure ulcers becomes necessary, adding a substantial burden and impacting maternal QOL.

Kaya et al [17] reported a higher prevalence of musculoskeletal disorders among caregivers of children with cerebral palsy compared to those of healthy children. Although this study did not identify specific contributing factors, it is conceivable that conditions like lower back pain in mothers would exacerbate their burden, significantly affecting their QOL. Other research [18] highlighted the cumulative fatigue characteristics among mothers raising children with disabilities who require home medical care. These mothers experienced higher levels of cumulative fatigue than the general female population, with no seasonal or temporal variations, and many consistently reported higher average values throughout the year than the average complaint rate among the general female population. Therefore, comprehending caregivers’ medical history and fatigue status is crucial, and there is a need for more research on interventions to improve caregivers’ physical functioning in the future [19].

While other studies have investigated items related to medical care, little focus has been given to positional changes. Compared with the use of ventilators or oxygen inhalation, the significant impact on QOL may be less apparent. However, position changes require care approximately every 2 hours, including during the night, leading to significant time constraints and the inability to secure adequate sleep. Consequently, positional changes have a substantial impact on QOL. It is essential to consider this when discussing necessary support for the child and family, and such discussions should be conducted collaboratively with the family.

### Support Toward Improving the QOL of Mothers of Children Requiring Medical Care at Home

The findings of this study show that the QOL of mothers caring for children requiring medical care at home is related to whether the child attends school or daycare, the duration of home care, and the necessity for positional changes. In addition, it was associated with a sense of caregiving burden. Caregivers must comprehend these factors as related elements and focus on collecting relevant information to consider the necessary support. However, previous studies have reported that services are still insufficient in this regard [6,13].

In 2021, a law was enacted to support children requiring medical care and their families [20]. This law explicitly identifies children requiring medical care as a legal category that enables structured support. Measures, such as the placement of nurses in daycare centers and schools to accommodate these children, as well as subsidy systems, have been established, which are expected to enhance the caregiving environment for children and reduce the caregiving burden on families. Similar to Japan, Caicedo [21] noted that the transition to home care is progressing in the United States, and support systems are needed. The study reported that among 3 groups—home care only, home care with the use of daycare, and institutional care—the home care–only group faced significant threats to physical and mental health [21], underscoring the substantial burden of home care alone. This finding aligns with trends in South Korea and Japan; mothers typically bear the sole responsibility for caring for children with disabilities [22], reflecting a global recognition of the substantial burden on these mothers and the need for robust support systems. Sharing and adapting support strategies across countries is crucial to provide effective assistance to mothers in each nation. Suzuki et al [23] reported that care coordination by nurses could alleviate the burden on parents resulting from home medical care for children who require medical devices. Using various support systems and having nurses assume care coordination roles are anticipated to further reduce the burden on parents, improving their QOL and overall well-being.

Regarding the QOL of mothers based on the condition of their children, a study on the QOL of mothers with children with attention-deficit/hyperactivity disorder [24] reported that the mother’s QOL was not associated with the child’s inattentiveness or hyperactivity symptoms as evaluated by the mother but with factors related to the mother and family, including the mother’s own inattentiveness, hyperactivity, depressive symptoms, perceived family support, and living conditions. Similarly, this study found that the presence of multiple medical conditions in children requiring medical care, intellectual developmental delays, and the need for severe care such as mechanical ventilation or oxygen therapy did not significantly correlate with the mother’s QOL. This aligns with previous research, suggesting that the child’s symptoms are not necessarily the most significant factors affecting the mother’s QOL, which caregivers must comprehend. However, previous research has indicated that the more severe a child’s functional limitations, the more likely the mother is to experience depression [25]. It has been reported that parents who care for children with tracheostomies at home have low QOL scores and show moderate levels of distress [26]. This suggests that a child’s condition can have a significant impact on the mother and that it is crucial to recognize the complexity of assessing a mother’s QOL based solely on the child’s condition. In order to provide high-quality care coordination for the family with children with a medical complexity, care coordinators need to pay attention to the evaluation and care planning of the entire family, especially the mother, who is the main caregiver in Japan [27]. Few countries take into account the opinions of families when formulating policies and national frameworks for providing care for children who use long-term ventilators [28]. It is important to properly assess the QOL of mothers, support them while taking into account the opinions of their families, and consider support for the whole country.

### Limitations

One of the limitations of this study was the small sample size, which limited the statistical power of the analysis.

Second, the adjusted *R*^2^ value of the multiple regression analysis was 0.327, indicating that the related factors did not fully elucidate the variance in the results, suggesting that other factors should be explored.

To address the disadvantages of a prior fragmented administration across different ministries, the Children and Families Agency was established in 2023, focusing on supporting children and their families. Given that this study was conducted before the law was enacted, it can serve as a reference for future comparative studies.

### Conclusions

Factors related to the QOL of these mothers included the presence of daycare or school attendance (β=.274), the length of home care (β=.305), and the need for postural changes (β=.41). The need for postural changes was found to have the most significant effect.

## Abbreviations

QOL: quality of life
WHO: World Health Organization
WHOQOL-26: World Health Organization Quality of Life-26
