# Correction: Factors That Affect the Quality of Life of Mothers Caring for Children With Medical Needs at Home: Cross-Sectional Questionnaire Study

**DOI:** 10.2196/79368

**Published:** 2025-06-25

**Authors:** Kanako Nakamura, Yuko Hamada, Ayaka Fujita, Seiichi Morokuma

**Affiliations:** 1 Fukuoka Jo Gakuin Nursing University Koga City Japan; 2 Department of Nursing Faculty of Nursing Shimonoseki City University Shimonoseki Japan; 3 Cicely Saunders Institute of Palliative Care Policy & Rehabilitation, Florence Nightingale Faculty of Nursing Midwifery & Palliative Care, King's College London London United Kingdom; 4 Department of Health Sciences Graduate School of Medical Sciences Kyushu University Fukuoka City Japan

In “Factors That Affect the Quality of Life of Mothers Caring for Children With Medical Needs at Home: Cross-Sectional Questionnaire Study” (Asian Pac Isl Nurs J 2024;8:e63946), the authors made the following corrections.

1. In Table 1, the data in the “Value, n (%)” column for the “Home medical care period (years)” category had been originally reported as follows:

<5: 19 (41)

5 or more: 25 (54)

This has now been corrected to:

<5: 20 (43)

5 or more: 26 (57)

Similarly, the data in the “Value, n (%)” column for the “Hospitalization” category had been originally reported as follows:

Yes: 27 (59)

No: 19 (41)

This has been corrected to:

Yes: 19 (41)

No: 27 (59)

2. In Table 2, in the “Home medical care period (years)” category, the values for the “Participants (N=46), n (%)” column had been originally reported as:

<5: 19 (41)

5 or more: 25 (54)

This has now been corrected to:

<5: 20 (43)

5 or more: 26 (57)

Furthermore, in the “Home medical care period (years)” category, the values for the “WHO QOL26 score” column had been originally reported as follows:

<5: 2.94

5 or more: 3.34

This has now been corrected to:

<5: 3.32

5 or more: 2.97

Similarly, in the “Hospitalization” category, the values for the “Participants (N=46), n (%)” column had been originally reported as:

Yes: 27 (59)

No: 19 (41)

This has been corrected to:

Yes: 19 (41)

No: 27 (59)

Furthermore, in the “Hospitalization” category, the values for the “WHO QOL26 score” column had been originally reported as follows:

Yes: 3.03

No: 3.24

This has been corrected to:

Yes: 3.24

No: 3.03

3.　Due to corrections to data related to the “Home medical care period (years)” subcategories in Tables 1 and 2, the following corrections have been made to the section “Attributes of Mothers and Children and Their QOL.”

The following sentence:

The WHOQOL-26 scores were significantly higher for the group with daycare or school attendance (P=.03) and for those with a home care duration of 5 years or more (P=.005).

Has been revised to:

The WHOQOL-26 scores were significantly higher for the group with daycare or school attendance (P=.03) and for those with a home care duration of less than 5 years  (P=.01).

The sentence below (last sentence of the “Attributes of Mothers and Children and Their QOL” section) has been deleted:

Regarding the duration of home care, while the WHOQOL-26 scores were higher for those with a duration of 5 years or more (P=.005), the physical and environmental QOL scores were higher for those with a duration of less than 5 years (both P=.007).

4.　 The first paragraph in the “Duration of home care” section was originally published as:

With regards to the duration of home care, significantly higher WHOQOL-26 scores were obtained in the group with a duration of 5 years or more. It is possible that through continued long-term home care, there is stabilization of the child’s health condition as a result of improvement in the child’s growth and the family’s management abilities. In their study on factors influencing the empowerment of mothers of children with disabilities during home care, Noguchi and Ohmachi [14] reported that a longer duration from diagnosis was significantly associated with higher levels of maternal empowerment. In this study, it is considered that the duration of home care, which correlates with the length of time from diagnosis, is related to a high level of maternal empowerment, resulting in significantly higher WHOQOL-26 scores in the group with a duration of 5 years or more of home care. Baker and Claridge [15] stated that numerous mothers found the transition period post their child’s diagnosis to be very difficult and stressful. However, most families were able to establish new daily routines and felt that they could manage their children’s illness. Therefore, it is believed that creating a life together as a family with a child receiving medical care may lead to stabilization in life. These findings suggest that patients with longer periods of home care may ultimately experience improvements in their QOL.

This has been replaced with the following text.

During home care, the WHOQOL-26 scores were significantly higher in the group with less than 5 years of care. Parents of children requiring medical care face additional burdens beyond typical childcare, including the technical aspects of medical care and various challenges, leading to a more demanding situation. Nygård and Clancy [14] reported, “When the burden of care becomes overwhelming, parents may lose motivation to continue caregiving, potentially leading to a decline in their caregiving abilities. This may affect the health of parents, family functioning, and the potential health status of children with illnesses.” As reported, this suggests that longer care periods may be associated with various impacts and a potential decrease in WHOQOL-26 scores in the group with care periods of five years or more. This finding is consistent with the results of this study. Moyes et al [15] reported that the need for support among parents of children requiring complex medical care changes over time.　As the duration of a child's home care increases, it is not that mothers' needs for support disappear, but rather that their needs change over time and they continue to seek support. In this study as well, it is considered that insufficient support tailored to the needs of mothers with longer home care periods may have contributed to the decline in WHOQOL-26 scores.

5. The following paragraph (last paragraph of “Duration of home care” section) has been deleted:

However, it should be noted that this study targeted young mothers ranging from 20 to 40 years of age, while the children’s ages were aged 12 years and younger. Considering the possibility of a lighter body weight during growth and development, mothers might not perceive home care as a significant burden. Research on the duration of home care is limited, and further studies on these factors are needed.

6. Finally, references [14] and [15] have been revised to the following:

14. Nygård C, Clancy A. Unsung heroes, flying blind-A metasynthesis of parents' experiences of caring for children with special health-care needs at home. J Clin Nurs. Aug 2018;27(15-16):3179-3196.

15. Moyes A, Abbott T, Baker S, Reid C, Thorne R, Mörelius E. A parent first: Exploring the support needs of parents caring for a child with medical complexity in Australia. J Pediatr Nurs. 2022;67:e48-e57.

The correction will appear in the online version of the paper on the JMIR Publications website, together with the publication of this correction notice. Because this was made after submission to PubMed, PubMed Central, and other full-text repositories, the corrected article has also been resubmitted to those repositories.

## References

[1] Nygård Carina, Clancy Anne (2018). Unsung heroes, flying blind-A metasynthesis of parents' experiences of caring for children with special health-care needs at home. J Clin Nurs.

[2] Moyes Anita, Abbott Theresa, Baker Sue, Reid Carlton, Thorne Rayleen, Mörelius Evalotte (2022). A parent first: Exploring the support needs of parents caring for a child with medical complexity in Australia. J Pediatr Nurs.

